# National Trends in Use of and Spending on Oral Anticoagulants Among US Medicare Beneficiaries From 2011 to 2019

**DOI:** 10.1001/jamahealthforum.2021.1693

**Published:** 2021-07-23

**Authors:** Aaron Troy, Timothy S. Anderson

**Affiliations:** 1 NYU Grossman School of Medicine, New York City, New York; 2 Department of Medicine, Beth Israel Deaconess Medical Center, Boston, Massachusetts; 3 Division of General Medicine, Beth Israel Deaconess Medical Center, Boston, Massachusetts; 4 Richard A. and Susan F. Smith Center for Outcomes Research in Cardiology, Beth Israel Deaconess Medical Center, Boston, Massachusetts; 5 Harvard Medical School, Boston, Massachusetts

## Abstract

This cross-sectional study examines patterns of use of direct oral anticoagulants and their association with Medicare spending.

## Introduction

Direct oral anticoagulants (DOACs) reached the market in 2010 and are associated with lower bleeding risks and decreased monitoring compared with warfarin, but also higher costs.^1^ Early adoption of DOACs was brisk, but to our knowledge, little is known about recent patterns of oral anticoagulant use and how the introduction of competing DOACs have affected Medicare spending.^2^

## Methods

We used the Medicare Part D Prescription Drug Event file, which includes annual aggregate data for all Medicare Advantage and stand-alone Part D plans, to study oral anticoagulant use between 2011 and 2019.^3^ Total spending and the number of beneficiaries who filled at least 1 prescription each year were examined for each anticoagulant and subclass. To compare trends in drug costs, we estimated annual inflation-adjusted costs per beneficiary for 1 year of treatment at atrial fibrillation dosing. Manufacturer discounts were estimated using the brand-name summed-discounts approach (eMethods in the Supplement).^4^ The study followed the Strengthening the Reporting of Observational Studies in Epidemiology (STROBE) reporting guidelines for reporting of cross-sectional studies. The study used only publicly available data and was determined to be exempt from Beth Israel Deaconess Medical Center institutional review board review.

## Results

**Figure 1.**
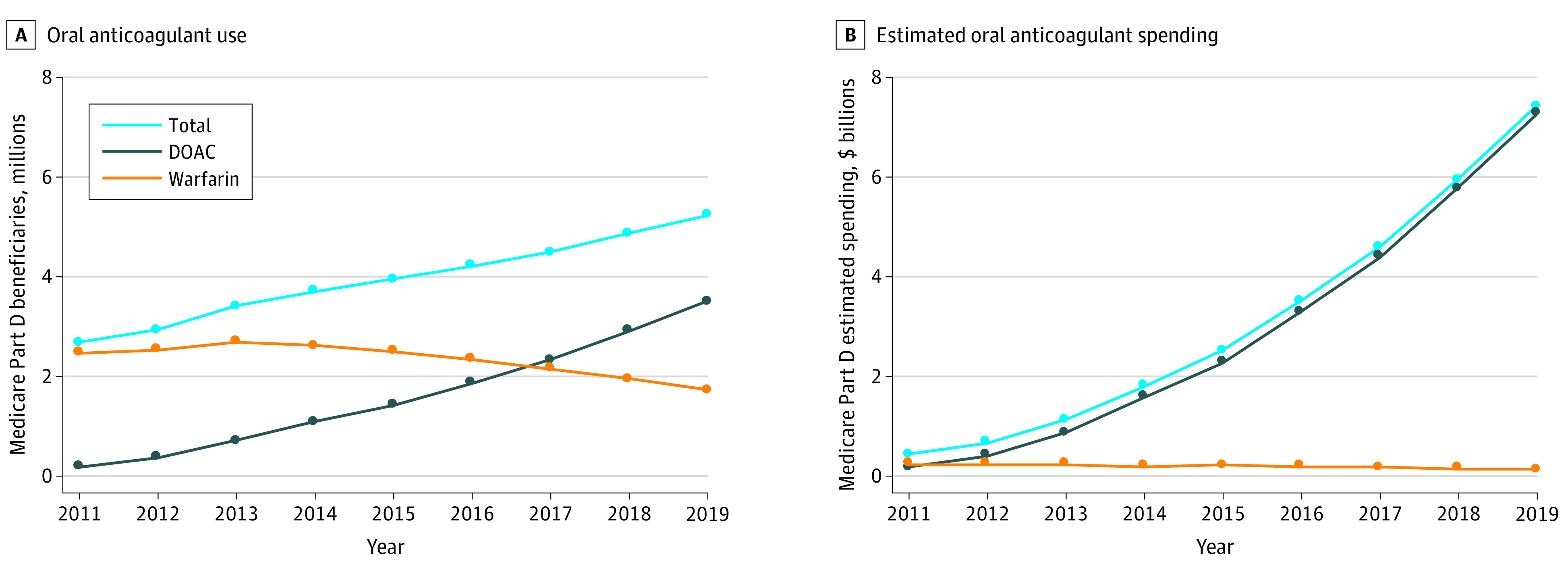
Oral Anticoagulant Use and Estimated Spending in Medicare Part D From 2011 to 2019 All prices presented in 2019 dollars after adjustment using the Consumer Price Index. Spending per year includes Medicare, plan, and beneficiary payments, accounting for branded medication discounts using the brand-name summed-discounts approach (eMethods in the Supplement).^4^ DOAC indicates direct oral anticoagulant.

Between 2011 and 2019, the number of Part D beneficiaries using oral anticoagulants increased from 2 681 919 to 5 241 483, or 9.2% to 11.5% of total beneficiaries (Figure 1A^4^). Of beneficiaries who used oral anticoagulants, the proportion using DOACs increased from 7.4% in 2011 to 66.8% in 2019, with an increase in DOAC users from 0.20 million to 3.50 million and a decrease in warfarin users from 2.48 million to 1.74 million.

Oral anticoagulant spending increased from $0.44 billion (0.6%) of overall Part D spending in 2011 to $7.38 billion (5.9%) in 2019 (Figure 1B^4^). In 2019, $7.23 billion was spent on DOACs and $0.15 billion was spent on warfarins. In 2019, the 3 most used medications were apixaban (41.4% of use; 59.5% of spending), generic warfarin (29.6% of use; 1.6% of spending), and rivaroxaban (21.6% of use; 33.2% of spending).

**Figure 2.**
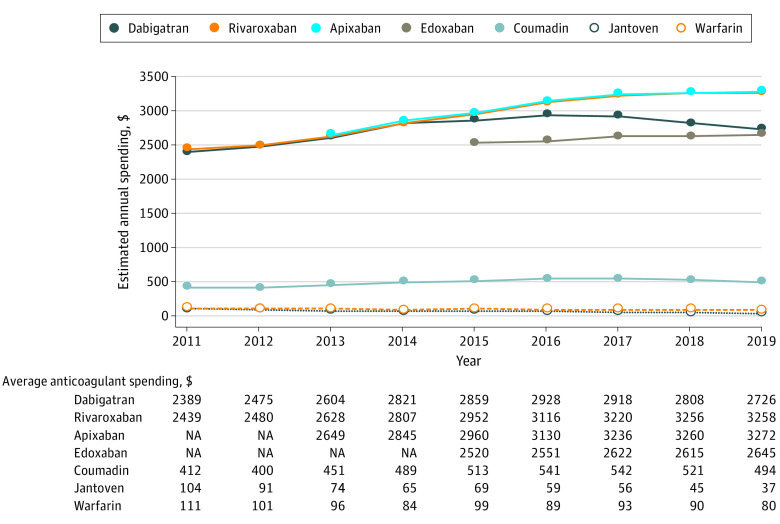
Estimated Annual Medicare Part D Spending on Anticoagulation per Beneficiary With Atrial Fibrillation From 2011 to 2019 All costs presented in 2019 dollars after adjustment using Consumer Price Index. Estimated total per beneficiary annual spending calculated as total Medicare spending per dosage unit multiplied by 365 days and by the number of pills per daily dose based on standard nonvalvular atrial fibrillation dosing. Spending per year includes Medicare, plan, and beneficiary payments, accounting for branded medication discounts using the brand-name summed-discounts approach (eMethods in the Supplement).^4^ NA indicates not applicable.

The estimated annual cost to treat 1 beneficiary with atrial fibrillation increased for all DOACs during the study period (Figure 2). Apixaban and rivaroxaban exhibited nearly identical cost growth, with average annual increases of 9.3% for apixaban and 9.5% for rivaroxaban. For generic warfarin, annual estimated costs decreased by 27.6% over the study period.

## Discussion

Since 2011, Medicare spending on oral anticoagulants has increased 16-fold to more than $7 billion annually, accounting for 5.9% of Part D spending in 2019. Spending growth was driven by rising Part D enrollment, increased oral anticoagulant use, a shift from warfarin to DOACs, and rising DOAC costs despite the introduction of 4 competing products.

This study had several limitations. It lacked patient-level data, which precluded stratifying the analysis by indication and duration of therapy. As total spending reported by Medicare does not account for manufacturer rebates, we estimated discounts using the brand-name summed-discounts approach, which provides an overall, but not drug-specific, annual discount rate and is subject to error.^4^ Spending estimates do not account for monitoring expenses, which are likely to be higher for warfarin but have been estimated at less than $1000 annually, nor does it account for patients’ time spent on monitoring. Thus, updated cost-effectiveness analyses comparing oral anticoagulants in the US are warranted.^5^

While higher prices for novel therapeutics like DOACs, which offer clear benefits, such as decreased drug-drug interactions^1^ and improved persistence, may partly reflect value and help drive innovation, the patterns and effects of spending on novel medications still merit attention. Competition among branded DOACs did not substantially curb annual spending increases, suggesting a lack of price competition, which is consistent with trends observed in other therapeutic categories.^6^ The exception was dabigatran, which faces a substantial disadvantage as the only DOAC requiring multiple doses per day, and whose price fell slightly from 2017 onwards. Use of generic DOACs, set to be released in coming years, as well as policy solutions, such as Medicare price negotiation, will be necessary to strike a balance between rewarding pharmaceutical innovations while ensuring competition helps prevent untenable Medicare drug spending.

## Notes

Supplement. eMethods.
