# Supplementary material for: National Trends in Use of and Spending on Oral Anticoagulants Among US Medicare Beneficiaries From 2011 to 2019
Source: JAMA Health Forum. 2021 Jul 23;2(7):e211693. doi: 10.1001/jamahealthforum.2021.1693 (PMC8796936; doi:10.1001/jamahealthforum.2021.1693)
Supplement: Supplement. — eMethods. [file jamahealthforum-e211693-s001.pdf]

Supplemental Online Content

Troy A, Anderson TS. National trends in use of and spending on oral anticoagulants among US Medicare beneficiaries from 2011 to 2019. *JAMA Health Forum*. 2021;2(7):e211693. doi:10.1001/jamahealthforum.2021.1693

eMethods.

This supplemental material has been provided by the authors to give readers additional information about their work.

© 2021 Troy A et al. *JAMA Health Forum.*

eMethods

To compare trends in drug costs over time, we estimated annual inflation-adjusted costs per beneficiary for one year of treatment at atrial fibrillation dosing, accounting for estimated rebates using a brand-name summed-discounts approach.[4] This approach consisted of four steps:

First, Medicare spending on individual dose units of oral anticoagulants were converted to costs per beneficiary for one year of treatment at atrial fibrillation dosing. This was done by multiplying spending per dosage unit by 365 days and by the number of pills per daily dose, using standard dosing for nonvalvular atrial fibrillation treatment (2 tablets per day for dabigatran and 1 tablet per day for all other anticoagulants).

Second, all costs were adjusted for inflation and presented in 2019 dollars after adjustment using the Consumer Price Index.

Third, the brand-name summed-discounts approach was used to estimate annual rebates and other discounts received by Medicare Part D for all branded medications.[4] This approach uses public data from Medicare Trustee and Office of the Inspector General reports to estimate overall Medicare Part D brand-name spending annually and then estimates the % brand-name discounts as the proportion of overall discounts divided by the proportion of annual spending spent on brand-name medications. Annual gross Part D spending was obtained from the CMS Medicare Part D Drug Spending Dashboard. Data on 2019 brand-name spending was not included in the 2021 Medicare Trustee reports. However, as the Bipartisan Budget Act of 2018 instituted an increase in pharmaceutical manufacturers' discounts in the Coverage Gap Discount Program from 50% to 70%, the impact of this change on pharmaceutical manufacturer spending was estimated in a 2018 Milliman White Paper, entitled "How will the Bipartisan Budget Act of 2018 impact Part D in 2019 and Beyond?", as leading to an additional $1.9 billion on coverage gap discounts by pharmaceutical manufacturers.

Year	Gross overall Part D spending	Estimated brand-name spending	Estimated manufacturer brand-name rebate and other discount spending	Brand-name % rebates and other discounts	Part D spending on oral anticoagulants	Part D spending on oral anticoagulants, after applying brand-name rebates and other discounts
2011	84.9	58.0	12.1	20.9%	0.5	0.4
2012	89.8	61.0	13.2	21.7%	0.8	0.7
2013	103.7	73.0	17.7	24.2%	1.4	1.1
2014	121.4	88.6	22.5	25.4%	2.4	1.8
2015	137.3	103.0	30.9	30.0%	3.5	2.5
2016	146.2	111.0	34.8	31.4%	5.0	3.5
2017	154.8	117.6	39.7	33.8%	6.8	4.6
2018	168.1	131.1	48.9	37.3%	9.4	5.9
2019	183.2	144.7	57.7	39.9%	12.2	7.4

Note: All spending figures in billions $. Overall discounts applied to branded medications only.

© 2021 Troy A et al. *JAMA Health Forum.*

Fourth, the estimated brand discounts were applied to the annual costs for each branded oral anticoagulant calculated in steps 1 and 2 to obtain the estimated after-rebate annual spending per year of treatment, and to the overall Medicare Part D annual spending estimates for each oral anticoagulant.

© 2021 Troy A et al. *JAMA Health Forum.*
